# Answering the Min-Cost Quality-Aware Query on Multi-Sources in Sensor-Cloud Systems †

**DOI:** 10.3390/s18124486

**Published:** 2018-12-18

**Authors:** Mohan Li, Yanbin Sun, Yu Jiang, Zhihong Tian

**Affiliations:** Cyberspace Institute of Advanced Technology, Guangzhou University, Guangzhou 510006, China; limohan@gzhu.edu.cn (M.L.); sunyanbin@gzhu.edu.cn (Y.S.); jiangyu@gzhu.edu.cn (Y.J.)

**Keywords:** sensor-based systems, sensor-cloud systems, data quality, quality-aware query, source quality

## Abstract

In sensor-based systems, the data of an object is often provided by multiple sources. Since the data quality of these sources might be different, when querying the observations, it is necessary to carefully select the sources to make sure that high quality data is accessed. A solution is to perform a quality evaluation in the cloud and select a set of high-quality, low-cost data sources (i.e., sensors or small sensor networks) that can answer queries. This paper studies the problem of min-cost quality-aware query which aims to find high quality results from multi-sources with the minimized cost. The measurement of the query results is provided, and two methods for answering min-cost quality-aware query are proposed. How to get a reasonable parameter setting is also discussed. Experiments on real-life data verify that the proposed techniques are efficient and effective.

## 1. Introduction

The quality of data can severely impact the data-driven applications. It is reported that data error rates of enterprises can be as high as 30% [1]. In some multi-sources applications, such as wireless sensor networks, Internet of Things or data fusion, data quality-aware sensing have been identified as one of the key concerns of sensor-based architecture [2]. In sensor-cloud systems which integrate hybrid networks [3], virtual sensors and their data can be queried by users via user interfaces. However, the quality of data returned by different data sources (i.e., virtual sensors) tends to be different due to different environments, and the quality of the historical data can be used as an index for generating plans of future queries. In some recent architectures, such as fog computing [4,5,6], edge devices are used to carry out a substantial amount of computation. The differences of computing capability and storage capacity between the devices also result in different quality of the data.

In these systems, a common fact is that there might be multiple sources providing the data of the same object, but the sources vary in data quality. Therefore, it is important to control and evaluate data quality. The quality of data and sources can be evaluated in the cloud based on classic data quality methods (which are not lightweight and therefore may be difficult to perform on sensors). Then, a set of data sources, which can provide relatively high quality data at low cost, will be selected to return the required data. In this way, we do not have to visit or wake up the rest of the data sources, and thus can save resources. Following example demonstrates the case of data sources with different data quality.

**Figure 1:**
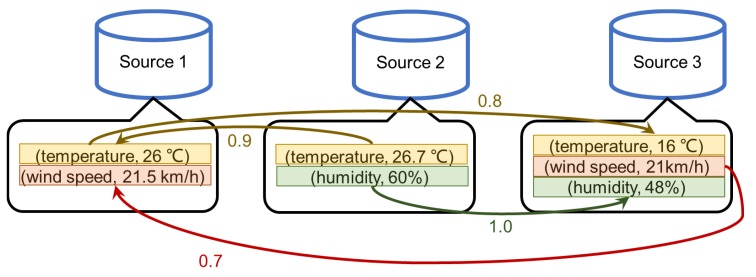
Data sources with different data quality.

**Example** **1.** *Three sources in Figure 1 provide weather conditions observed in the same place at the same time. However, these sources provide different observations of the same object. For instance, Source 1 claims that the temperature is 26 °C, but Source 2 and Source 3 claim that the temperature is 26.7 °C or 16 °C.*

*Some relative quality constraints can be used to infer the relative quality of the observations. For example, if we know that the temperature sensor used by Source 2 possibly be a newer model of the sensor of Source 1, we can infer that the value of temperature provided by Source 2 tends to be more accurate than Source 1. Arcs with probability can represent the existence of relative quality constraints. In Figure 1, the arc from (temperature, 26.7 °C) to (temperature, 26 °C indicates that there may exist a relative quality constraint that “the sensor of Source 2 provides higher quality temperature than the sensor of Source 1”, and the probability of the existence of the constraint is 0.9.*

*According to relative quality constraints shown by Figure 1, the observations of temperature and wind speed of Source 3 might be less accurate than those of Source 1 and Source 2. That is, if we only access the data from Source 3, we might get inaccurate values. In this case, if the user is willing to pay a higher cost, perhaps we can access the other two data sources to obtain higher quality data.*

When selecting sources for answering a query with relative quality constraints, both low cost and high quality are required. In this paper, we study the min-cost quality-aware query (MQQ for short) answering problem which aims to get high quality results from multi-sources with the minimized cost. This paper is extended based on the conference version of SCS 2018 [7]. The contributions of this paper are as follows.

1. A general definition of MQQ answering problem is provided.
2. The data quality measurement of quality-aware query on multi-sources is defined based on relative data quality constraints. Two methods for solving the min-cost quality-aware query answering problem are proposed.
3. If the users do not have enough knowledge of the data, it is often difficult for them to set the reasonable quality threshold. To deal with this problem, a method is proposed to automatically help the users to find a proper quality threshold.
4. The experiments on real-life data are conducted, which verifies the efficiency and effectiveness of the provided solutions.

The rest of this paper is organized as follows. Section 2 discusses the related work. Section 3 provides the problem definition. Section 4 gives the data quality measurement. Section 5 studies the solutions of MQQ answering problem. Section 6 discusses the strategies of setting a reasonable quality threshold. Section 7 shows the experimental results. Section 8 concludes the paper.

## 2. Related Work

**Data quality.** There is currently a lot of work on constraint-based data quality [8,9,10,11,12,13,14,15,16,17,18,19,20,21,22]. The problem of data quality evaluation is studied in these works. These constraint-based methods can be roughly categorized into two classes.

(1) The first class of methods can directly compute the quality score of a data set. Some constraints, such as functional dependencies, conditional functional dependencies, denial constraints and editing rules, are used as data quality rules to detect errors in a given dataset [8,9,10,11]. Some systems use these kinds of rules to find the dirty data in a given data set [12], and the quality score of a data set can be defined as the proportion of clean data in the data set. Meanwhile, the trustworthy of data sources in data fusion can also be considered as the quality score of data sources [13].

(2) The second class consists of methods for computing relative data quality. The relative quality has been studied for many years, and a lot of rules are proposed to evaluate the relative completeness, accuracy, currency, etc. The relative data quality constraints can be derived based on their methods. For example, the relative accuracy constraints can determine the relative accuracy of different tuples [14]. Inclusion dependencies and conditional inclusion dependencies can determine the relative completeness of data set [15]. The currency rules can be used to determine the relative currency of different tuples or data sources [16,17]. Some techniques for data fusion and truth discovering also study how to derive relative data quality constraints [18,19,20]. These techniques can find the copy dependencies between different sources, and the copy dependencies can be used to derive the relative currency or completeness constraints of different sources [21,22].

The two classes of methods can either directly compute the absolute quality score of one source or compute the relative quality score between two sources based on the observations. However, they do not take into consideration how to evaluate and guarantee the data quality of query results, and thus is different from our work.

**Sensor-cloud.** There are currently many studies on the architecture of sensor-cloud [3,23,24,25,26,27,28,29]. Alamri A. et al. [3] surveyed the early works of sensor-cloud. Madria S. et al. [23] proposed a new cloud of sensor (CoS) which was composed of virtual sensors built on top of physical wireless sensors rather than the device of traditional cloud. Based on the CoS, Olympus [24] is proposed by combining an information fusion and CoS-based decentralized WSAN virtualization model. Application decision processes are performed partly within physical sensors and partly within the cloud. Fazio M. et al. [25] proposed a cloud framework called Cloud4sens, which combines both data-centric and device-centric models to meet different needs of cloud clients. To improve the performance of cloud-based sensor system, Lyu Y. et al. [26] proposed a periodic-task based high-performance scheduling model with a cloud-supported caching mechanism for the multisensor gateway. Abdelwahab S. et al. [27] proposed a global architecture of Cloud of Things, and adopted a distributed sensing resource discovery and virtualization algorithms to deploy virtual sensor networks on top of a subset of the selected IoT devices. Zhu C. [28] proposed a multi-method data delivery scheme for green sensor-cloud among WSNs, cloud and users to achieve low delivery cost and less delivery time. Dinh T. et al. [29] proposed a location-based interactive model of IoT-cloud for mobile cloud computing applications, and the model provides sensing services on demand based on interests and locations of mobile users.

A common feature of these sensor-cloud architectures is that they combine some of the hybrid sensor networks to get a virtual sensor network, while using cloud services to handle the interaction of the virtual sensor network and end users. This ensures that the underlying hybrid sensor networks are transparent to the end users. The evaluation of data quality will consume a lot of computing resources (some evaluation problems require square or higher time overhead, or even be NP-hard problems), so it is difficult to be taken on the sensor side, and we need to deploy it in the cloud. There are three benefits. First, we can utilize the computing power of the cloud to quickly complete the evaluation of data quality. Second, the deploying it in the cloud can make the quality evaluation independent of the specific architecture of the underlying virtual sensor network. Third, since we will select better sensors to answer user queries after the evaluation of data quality, we can also save computing resources and power on the sensor side.

**Quality-aware sensing.** There are a lot of works to ensure some extra properties when executing queries in data-driven and sensor-based systems, such as security [30,31,32,33,34] or trustiness [35,36,37]. Quality-aware query has also been studied in some data-driven systems [38,39,40]. Lian X. et al. [38] formalized the RDF data by inconsistent probabilistic RDF graphs, and studied the quality-aware subgraph matching problem. Yeganeh N. K. et al. [39] provided a data quality-aware system architecture which supports data quality profiling and data quality-aware SQL. The sample conditional data profile generated by the system can be used as a type of quality constraints in our work, but the work does not focus on how to get a best query result with cost and quality requirements. Wu H. et al. [40] studied the quality-aware query scheduling in wireless sensor networks. The provided framework can determine the target quality of each query. The work can be used as the basis for this study, but it does not consider optimization problems when there are multiple query results, and it also does not consider how to do quality-aware query in sensor-cloud systems.

## 3. A General Definition of MQQ Answering Problem

### 3.1. Preliminaries

First we provide some basic concepts which will be referred in the following sections.

**Data sources.** Let $S=\{S_{1},\ldots ,S_{n}\}$ be a set of data sources, where $S_{i}=\left\{\phi_{1},\ldots ,\phi_{m_{i}}\right\}$ is the *i*th data source consisting of $m_{i}$ observations. An observation ϕ=(s,o,v) is a triple consisting of a source *s*, an object *o* and a corresponding value *v*. For instance, $\left(S_{2},humidity,60\%\right)$ indicates that Source $S_{2}$ observes that the humidity is 60%. All the observations in a same source is with the same *s*.

**Data quality constraints.** Quality function hq is defined to represent data quality constraints. $hq(\phi ,\phi^{\prime })=p$ means that the quality of ϕ probably be *higher* than the quality of $\phi^{\prime }$, and the existence probability of this constraint is *p*. For example, $hq(\phi ,\phi^{\prime })=0.9$ means that the data quality of ϕ is higher than $\phi^{\prime }$ with the probability 0.9. The quality constraints can be considered as the relative data accuracy or other relative data quality measurements [14,15,16,17]. These constraints can be used to evaluate the quality of the observations and sources. How to find and leverage these constraints will be addressed in the next section.

**Access cost.** Each data source $S_{i}\in S$ has a access cost $cost\left(S_{i}\right)\in R^{+}$, indicating the cost of querying the data provided by $S_{i}$. High data quality sometimes means high access cost, thus MQQ is defined to try to balance the cost and quality.

### 3.2. Definition of MQQ Answering Problem

**Quality-aware query.** A quality-aware query *Q* is a query with data quality requirements. *Q* is in the form of $Q=(O_{Q},\theta )$, which is a pair of an object set $O_{Q}$ and a quality threshold θ. $O_{Q}$ consists of the objects queried by *Q*. θ is a lower bound, which represents the requirement that the quality of the returned observations must be no less than θ.

**MQQ answering problem.** A MQQ aims to find the observations of $O_{Q}$ from sources in S which satisfying data quality lower bound and with minimized access cost. Formally, the MQQ answering problem can be defined as follows.

**Input:** a quality-aware query $Q=(O_{Q},\theta )$,

**Output:** a observation set Φ which satisfies the following conditions:

1. For each $o\in O_{Q}$, ∃ϕ∈Φ such that ϕ is the observation of *o*, that is, Φ contains the observations of all queried objects,
2. Φ can satisfy the quality lower bound θ,
3. $∄\Phi^{\prime }$ returned by $S_{\Phi^{\prime }}\subseteq S$ such that $\Phi^{\prime }$ satisfies condition (1) and (2), and $\sum_{S\in S_{\Phi^{\prime }}}cost(S)<\sum_{S\in S_{\Phi }}cost(S)$.

As of now, we have not discussed how to calculate the data quality of query results. In the following sections we will first consider how to calculate the data quality of a set of observations, and then present the analysis of MQQ answering problem.

## 4. Measuring Data Quality Based on Quality Constraints

Ideally, each data source corresponds to a certain quality score (sometimes the score is called absolute quality score), then we can use certain quality scores to directly resolve subsequent quality-aware query problems. However, in many cases, we can only get the relative quality rule of two data items [14,15,16,17]. For example, an accuracy rule $\psi =\forall t_{1},t_{2}\;\;\omega \to t_{i}{≺}_{A_{i}}t_{j}$ can enable us to determine the relative accuracy of tuple $t_{i}$ and $t_{j}$ on attribute $A_{i}$. A uncertain currency rule $\psi =\forall t_{1},t_{2}\;\;\left(\omega \to t_{i}{≺}_{A_{i}}t_{j},cer\left(\psi \right)\right)$ can provide the possibility that observations provided by one source will become obsolete earlier than another source. The semantic of a quality rule is that if the condition ω on the left-hand side is satisfied, the inequality of $t_{i}\left[A\right]$ and $t_{j}\left[A\right]$ on the right-hand side is established (with a probability or certainty). $t_{i}\left[A\right]$ and $t_{j}\left[A\right]$ are the values (i.e., observations) of two tuples on object *A*, thus the inequality of right-hand side indicate the relative data quality. Base on the rules, if two tuples satisfy ω, $hq(t_{i}\left[A\right],t_{j}\left[A\right])=p$ can be derived from the right-hand side, where $t_{i}\left[A\right]$ and $t_{j}\left[A\right]$ correspond to the ϕ and $\phi^{\prime }$ in the definition of data quality constraint in Section 3.1.

In practice, quality rules are either discovered from a training data set [41,42], or written by the domain experts. For example, the owner of the sensor network may provide the knowledge that “*model A is a newest model of the temperature sensor used in my network, which can provide more accurate data than other models in 90% of cases*”. Based on the knowledge, if we have a set of temperature data {26,27,28}, and we find that 27 is provided by a sensor of *Model A*, 26 and 28 is provided by sensors of *Model B*, we will know that hq((ModelA,temperature,27),(ModelB,temperature,26))=0.9.

To deal with the relative quality constraints, we propose a data source quality score based on the semantic of possible worlds.

### 4.1. Quality Graph

As defined before, quality constraints are represented by the quality function hq. $hq(\phi ,\phi^{\prime })=p$ indicates that the quality constraint exist with probability *p*. Thus, uncertain graphs [43,44] can be used to model the constraints, that is, observations are the nodes in the graph, and relative quality constraints are the weighted arcs.

**Definition** **1** (quality graph)**.** *Given an object o, the observation set $\Phi_{o}$ containing all the observations corresponding to o, and the data quality function corresponding to $\Phi_{o}$, the quality graph $G_{o}=\left(V,A\right)$ is defined as follows.*

*1.*  *Each node $v_{\phi }\in V$ corresponds to an observation in $\Phi_{o}$.*
*2.*  *For each pair $\left(\phi ,\phi^{\prime }\right)$, if $hq(\phi ,\phi^{\prime })\neq null$, there exists an arc from ϕ to $\phi^{\prime }$ in A, the weight of the arc $\left(\phi ,\phi^{\prime }\right)$ is $hq(\phi ,\phi^{\prime })$.*

**Figure 2:**
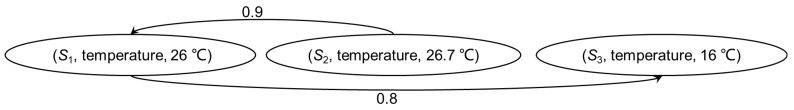
The direct graph of the observations of temperature in Figure 1.

**Figure 3:**
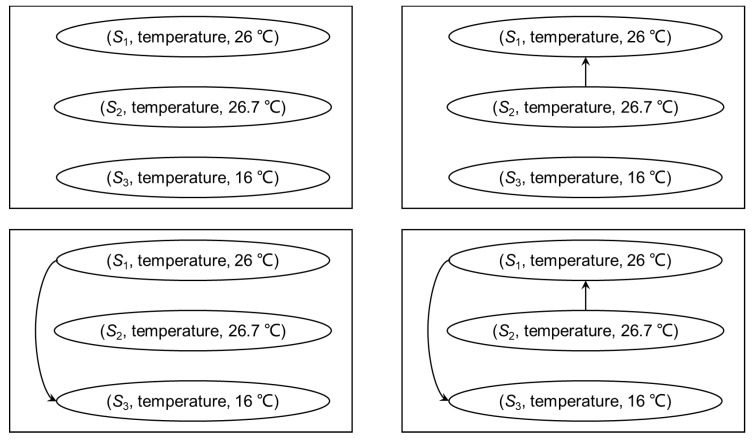
The possible worlds of a quality graph.

In the definition, $hq(\phi ,\phi^{\prime })=null$ means that hq is not defined at $\left(\phi ,\phi^{\prime }\right)$. Figure 2 is the quality graph of the object “temperature” in Figure 1. For the arcs in $G_{o}$, the semantic of the arc weight is the probability of the arc’s existence. For example, if the weight of an arc *a* is 0.9, the probability of *a*’s existence is 0.9, and the probability of non-existence is 1−0.9=0.1. Therefore, possible worlds [45] can be used to describe different situations of data quality. Figure 3 shows the the four possible worlds corresponding to Figure 2, and the probabilities of each possible worldc are 0.02, 0.18, 0.08, and 0.72, respectively.

### 4.2. Data Quality Score

The data quality score of an observation ϕ of the object $o_{\phi }$ is defined as the probability that the quality of ϕ is NOT lower than any other observations of $o_{\phi }$. We use $G_{o_{\phi }}$ to denote the quality graph of $o_{\phi }$. In any possible world, the quality of ϕ is higher than the quality of $\phi^{\prime }$ if there exists an arc from ϕ to $\phi^{\prime }$. Therefore, the quality score of ϕ is the probability that the in-degree of $v_{\phi }$ equals to 0, where $v_{\phi }$ is the node corresponding to ϕ.

**Definition** **2** (quality score of an observation)**.** *Let W be the set of all the possible worlds corresponding to $G_{o_{\phi }}$. $dq\left(\phi \right)=\sum_{w\in W}P_{w}\times f\left(w\right)$ is the quality score of *Φ*, where f(w)=1 if node $v_{\phi }$ has an in-degree of 0 in w, otherwise f(w)=0.*

According to the semantics of the arcs in the quality graph, a naive idea to calculate the quality score of a node is to enumerate all possible worlds and check in which possible worlds the node’s in-degree is 0. However, if we assume that the existence of the arcs are independent of each other, the quality score of ϕ is the product of the non-existence probabilities of all the in-arcs of ϕ, that is, $dq\left(\phi \right)=\prod_{a\in in(v_{\phi })}\left(1-weight(a)\right)$, where $in(v_{\phi })$ is the in-arc set of $v_{\phi }$, and weight(a) is the weight of arc *a* in $G_{o_{\phi }}$. Furthermore, we assume the observations are independent of each other, the quality of an observation set Φ can be similarly defined.

**Definition** **3** (quality score of observation set)**.** *Given an observation set *Φ*, the quality of *Φ* is the probability that for each observation ϕ∈Φ, the quality of ϕ is not lower than any other observations, that is,*

(1) $$dq\left(\Phi \right)=\prod_{\phi \in \Phi }dq(\phi ).$$

Given a quality-aware query $Q=(O_{Q},\theta )$, the query result of *Q* is a set of observations. Therefore, the data quality of the query results is the same as the quality of the observation set. More precisely, condition (2) (i.e., “Φ can satisfy the quality lower bound θ” ) in the definition of MQQ answering problem can be rewritten by “dq(Φ)≥θ”.

## 5. Methods for Solving MQQ Answering Problem

As is defined in Section 3, if Φ is a valid output of MQQ answering problem, Φ should satisfy three conditions, i.e., (1) Φ covers all the objects in $O_{Q}$, (2) dq(Φ) is no less than θ, and (3) the access cost of finding Φ is minimized. Please note that if condition (2) is not considered, MQQ answering the problem is an equal problem of “weighted set cover” which is a NP-hard problem [46].

In this section, we first provide a search and prune method to accurately solve the MQQ answering problem, then provide an approximate method to try to get a feasible (but not necessarily optimal) solution quickly.

### 5.1. The Search and Prune Method

The main idea is that we first find the observation set with highest quality score, then we continually replace one observation in current set by an unused observation of the same object with a lower or equal quality score, to see if the new set after replacement can satisfy the quality threshold at a lower cost. The replacement step is repeated until the quality threshold cannot be satisfied or there is no less-cost solution.

**Figure 4:**
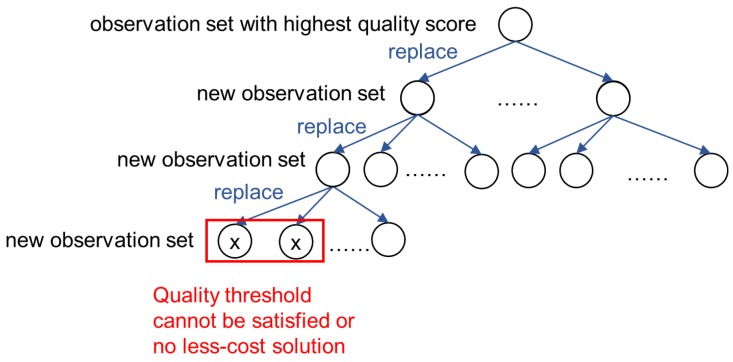
The main idea of the search and prune method for MQQ answering.

Figure 4 shows the search tree of this process. Each node in the tree represents an observation set, and each child corresponds to a substitution of its parent. Since we replace exactly one observation each time, for each node $\Phi =\{\phi_{1},\ldots ,\phi_{k}\}$ in the search tree, if $\Phi^{\prime }=\left\{\phi_{1}^{\prime },\ldots ,\phi_{k}^{\prime }\right\}$ is the child of Φ, $\Phi^{\prime }$ has exactly one observation different from Φ. That is, ∃j s.t. $\phi_{j}\neq \phi_{j}^{\prime }$, and $\phi_{i}$ is the same as $\phi_{i}^{\prime }$ for any other i≠j. Moreover, we have $dq\left(\phi_{j}\right)\geq dq\left(\phi_{j}^{\prime }\right)$.

To ensure that the search can finally enumerate all possible solutions, we sort all the observations associated with the same object based on their quality scores. The current observation is hereby replaced by the next observation in the sorted sequence to generate a new observation set in the search tree. For each node Φ in the search tree, Φ may have 0 to $|O_{Q}|$ child nodes. The total number of nodes in the full search tree is exponential, thus we need an effective pruning strategy to speed up the process.

**Prune strategy.** Since we always replace one observation by another observation with an equal or lower quality score, the quality score of a parent node is always not lower than its children. Theorem 1 formalizes this conclusion.

**Theorem** **1.** *For any node *Φ* and its child $\Phi^{\prime }$ in the search tree, $dq\left(\Phi \right)\geq dq\left(\Phi^{\prime }\right)$.*

**Proof.**  Let $\Phi =\{\phi_{1},\ldots ,\phi_{k}\}$ and $\Phi^{\prime }=\left\{\phi_{1}^{\prime },\ldots ,\phi_{k}^{\prime }\right\}$. As we have discussed before, ∃j s.t. $\phi_{j}\neq \phi_{j}^{\prime }$, $dq\left(\phi_{j}\right)\geq dq\left(\phi_{j}^{\prime }\right)$, and for any i≠j, $\phi_{i}$ is the same as $\phi_{i}^{\prime }$. According to the definition of quality score of observation set, $dq\left(\Phi \right)=dq\left(\phi_{j}\right)\times \prod_{i\neq j}\left(dq\left(\phi_{i}\right)\right)$, and $dq\left(\Phi^{\prime }\right)=dq\left(\phi_{j}^{\prime }\right)\times \prod_{i\neq j}\left(dq\left(\phi_{i}^{\prime }\right)\right)$. Therefore, $dq\left(\Phi \right)\geq dq\left(\Phi^{\prime }\right)$. ☐

For any node Φ and its child $\Phi^{\prime }$, $dq(\Phi^{\prime })\leq \theta$ if dq(Φ)≤θ. Thus, if the quality threshold θ cannot be satisfied by Φ, it also cannot be satisfied by any descendant node of Φ. Thus the subtree rooted at Φ can be pruned off.

Let $O_{Q}$ be the object set queried by *Q*, the time complexity of the worst case is still exponential, i.e., $O(|O_{Q}{|}^{\left|S\right|})$. It is still unbearable in the condition of high efficiency requirement. Therefore, we propose an approximate search strategy which can quickly provide a feasible solution when the user is willing to relax the requirements for the cost.

### 5.2. Approximate Search

When a specified data source is asked to return multiple observations, the access cost only needs to be paid once. Thus, a replacement that adds data sources necessarily reduces the quality and increases the cost at the same time. For node Φ and its child $\Phi^{\prime }$, we have Theorem 2, where $S_{\Phi }$ and $S_{\Phi^{\prime }}$ are the source sets returning Φ and $\Phi^{\prime }$ respectively, $cost\left(\Phi \right)=\sum_{S\in S_{\Phi }}cost\left(S\right)$ is the cost of Φ.

**Theorem** **2.** *$cost\left(\Phi \right)\leq cost\left(\Phi^{\prime }\right)$ and $dq\left(\Phi \right)\geq dq\left(\Phi^{\prime }\right)$ if $S\notin S_{\Phi }$ and $S_{\Phi^{\prime }}=S_{\Phi }\cup \left\{S\right\}$.*

**Proof.**  According to Theorem 1, $dq\left(\Phi \right)\geq dq\left(\Phi^{\prime }\right)$. Since $S_{\Phi^{\prime }}=S_{\Phi }\cup \left\{S\right\}$, $cost\left(\Phi^{\prime }\right)=\sum_{S^{\prime }\in S}cost(S^{\prime })+cost\left(S\right)=cost\left(\Phi \right)+cost\left(S\right)$, we have $cost\left(\Phi \right)\leq cost\left(\Phi^{\prime }\right)$. ☐

According to Theorem 2, an idea to quickly get a feasible solution is to drop the substitution that add new data sources. That is, for any node Φ, only the observations returned by $S_{\Phi }$ are used to do the replacement. More precisely, for any node Φ and its child $\Phi^{\prime }$, we stipulate that a replacement should ensure either $S_{\Phi^{\prime }}=S_{\Phi }$ or $S_{\Phi^{\prime }}=S_{\Phi }$\{S}, where $S\in S_{\Phi }$. The search is terminated when the quality threshold cannot be satisfied or there is no less-cost solution.

It is easy to observe that this search strategy only considers the subset of the data sources which provide the observations with the best quality score. The solution returned by this strategy necessarily satisfies the conditions (1) and (2) in the definition of MQQ answering problem, but does not necessarily satisfy the condition (3) (i.e., the cost may not be the lowest).

### 5.3. Discussions

Please note that the above search methods do not have much restrictions on the definition of data quality score. The only restriction is that the quality score should not reduce after replacement. More concretely, our data quality definition for the observation set is in a multiplicative form, but other forms (e.g., average or maximum) of data quality metrics which guarantee the quality score not reduce after replacement, are applicable to the search strategy in this section. If there are any other reasonable quality metrics which may not based on the semantic of possible worlds but can ensure that the quality score do not reduce after replacement, our search method can also be applied to these quality metrics with a few modifications.

When implementing the above two strategies, the efficiency can be further optimized in two aspects.

1. For the two search strategies, it is very important to efficiently get the next data source to do the replacement. To this end, |O| ordered lists can be maintained, and the *i*th ordered list stores the high-to-low ordering of the sources that can provide object $o_{i}$. A element of the ordered list is a quad of the form (S,ϕ,dq(ϕ),next), where next is a pointer to the next quad in the ordered list. Thus, during the search, we can use dq(ϕ) to calculate the quality score of the current search tree node, and use next to get the next data source that can be used to do the replacement in constant time.
2. Different branches of the search tree may have the same node. In implementation, we can first check to see if the current node already has a duplicate that has been processed. The node is discarded if the answer is yes. This operation can avoid repeating process and can save a lot of time.

## 6. A Method for Selecting the Quality Threshold

When writing a MQQ $Q=(O_{Q},\theta )$, if the end user does not have much knowledge about the data, it might be difficult to set a proper threshold θ. Therefore, we propose a method that can automatically determine the reasonability of a threshold, and can help the user to find a proper threshold.

### 6.1. Determining the Reasonability of a Threshold

There are two cases that the quality threshold is not set properly.

1. The quality threshold is set too high such that no observation set can satisfy the threshold.
2. The quality threshold is set too low, which is likely to cause large search space, and to lead to unendurable query latency.

Therefore, determining the reasonability of a threshold can be divided into two steps.

#### 6.1.1. Deal with High Threshold

First we need to determine whether the threshold is too high. A quality threshold θ is deemed to be too high if we cannot find any data source which can provide the queried observations and meet the quality threshold. This is a trivial problem since we just need to check whether the root node of the search tree can satisfy θ. As we have discussed in Section 5, the root node corresponds to the highest quality score. Therefore, all the nodes in the search tree cannot satisfy θ if the root node fail to satisfy θ. If the time to construct a search tree node can be considered as constant, then the time complexity to determine whether the threshold value is too high is O(1).

#### 6.1.2. Deal with Low Threshold

If the threshold is not too high, we need to determine whether it is too low. Since a low threshold will make the query to be processed slowly, the user should be reminded to raise the threshold in this case. We found that when using accurate search, the search time may rise by more than twofold when the threshold is decreased by 0.01 in some cases.

To determine whether the threshold is too low is much difficult. The first difficulty is that we cannot precisely define what is “too low”. To deal with this difficulty, we can use historical data to estimate the query latency and let the user to determine whether the threshold is too low. The second difficulty is that it is hard to precisely know the query latency without any prior knowledge since the data needed by different queries are also different. However, it is easy to observe that the query delay is mainly affected by the size of search space, thus the estimate of query latency can be converted to the estimate of the search space size.

**Estimate of the search space size.** We provide a method to estimate the size of search space based on the idea of estimating the layer number of the tree using depth-first search. The process is as follows. We expand the search tree from top to bottom in the way similar to Section 5, but the difference is that we do not generate a full search tree. We only do the replacement in each layer once, that is, generate only one new node in each layer. The process of the expanding stops if the new node cannot meet the quality threshold requirement. The height of the tree at the time point that the process stopped is the estimate of the search tree. For example, if we only generate the left most nodes in the search tree shown by Figure 4, the height of the tree is estimated to be 4 because the node generation of the search tree stops at the fourth layer. Let *h* denote the real height of the search tree, and $\hat{h}$ denote the estimated height of search tree, then the search space size (i.e., the upper bound of the total number of nodes of the search tree) can be estimated to be ${|O_{Q}|}^{\hat{h}-1}$ since each node has at most $|O_{Q}|$ children.

A correspondence table of search space size and time overhead can be maintained based on the historical data. Alternatively, other models of search space size and query latency also can be built based on some machine learning methods. Based on the models, the estimate of the search space size can be converted to the estimate of the query latency.

However, the accuracy of the estimate is determined by the accuracy of the estimate of the height of the search tree, and this estimate has a lot to do with the position of the nodes generated for each layer. For instance, in Figure 4, if we generate the rightmost (rather than the leftmost) node in each layer, the estimate of the height of the search tree is at least 5.

Since the accurate search needs to traverse all branches, it is not difficult to observe that we only generate a path in the search tree when doing the estimation. The real height *h* is the length of the longest path in the search tree, and the estimate of the height must be no more than the real height. Formally, we have

(2) $$\hat{h}\leq h.$$

Based on this conclusion, we can infer that the larger the estimate, the closer it is to the real height. To find a better estimate of the height, we propose two generation strategies for nodes, which are applicable to different scenarios.

**Strategy 1 (FixedPos).** This strategy generates the node in a fixed position each time. Concretely, a location *k* is selected before the estimation, and the *k*th data source is replaced to obtain a new child node each time. This strategy applies to situations where pruning is not considered and there is some domain knowledge of the query. For example, when we know in advance that “the *k*th object in the query has the most replaceable data sources”, searching down at position *k* is more likely (not necessarily) to get a higher search tree than other positions. $\hat{h}$ tends to be closer to *h*.

**Strategy 2 (RandPos).** This strategy generates the node in a random position each time. When pruning is considered or there is no domain knowledge of the query, if we do not perform an accurate search, we cannot know how to use depth-first search can get the most accurate estimate. Therefore, we can randomly selecte a $k_{i}$ ($0<k_{i}\leq \left|O_{Q}\right|$) for layer *i*, and the $k_{i}$th data source is replaced to obtain a new child node in layer i+1. If time permits, we can also perform multiple random strategies and pick the largest (and therefore the most accurate) estimate.

After $\hat{h}$ is obtained, the query latency can be estimated based on the table or model mentioned above. Then, the estimated query latency can be reported to the user, and the user can determine whether the latency is acceptable. If not acceptable, the threshold is marked as too low.

### 6.2. Finding a Reasonable Threshold

If the threshold is considered to be unreasonable, we can give some suggestions to help the user correct the threshold.

1. If the threshold is considered to be too high, we only need to return the quality score of the root node of the search tree and tell the user that there is no query result higher than this score.
2. If the threshold is considered to be too low, we need to return a set of candidates to guide the user in adjusting the threshold. As stated in Theorem 1, when the search tree is expanded to the lower layer, dq is not increased (i.e., the value of dq only decreases or keeps unchanged). Therefore, in the estimation process, the layer where dq changes can be recorded and converted into a query latency. That is, we ignore those layers whose dq is unchanged and record the layers whose dq decreases. The historical data can be used to learn the possible query latency regarding the number of layers. The recorded can be given to the user as a candidate set to help the user to adjust the threshold setting.

## 7. Experimental Results

We conduct the experiments on a real-life data set. The codes are written in Python and run on a machine with i5 2.50 GHz Intel CPU and 8 GB of RAM. The dataset consists of air quality data generated by a network of 56 sources located in Krakow, Poland. The dataset is from https://airly.eu/. The available object set is {temperature, humidity, pressure, pm1, pm25, pm10}. Since the data set does not provide the cost of data sources, we random assign each source a cost. In practical applications, the cost can be the bandwidth, power consumption, or the money of getting data, etc. We first compared the two different search methods (i.e., the accurate search and the approximate search). After that, we studied the performance of the two strategies of search space size estimation.

### 7.1. Performance of the Search Methods

#### 7.1.1. Varying |S|

**Figure 5:**
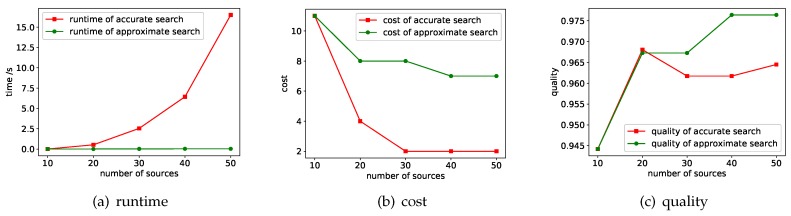
Experimental results when varying |S|.

A parameter that has a significant impact on efficiency and performance is the number of data sources, i.e., |S|. Figure 5 shows the experimental results of runtime, cost and quality under different |S|. Here we fixed $|O_{Q}|$ to 4 and the quality threshold to 0.95.

**Runtime.** We compared the runtime of accurate search and approximate search. The worst case of accurate search is $O(|O_{Q}{|}^{\left|S\right|})$. This theoretical analysis is consistent with the experimental results. The efficiency of approximate search is much higher than that of accurate search. When the number of data sources is 10, the time cost of the two is similar, but as the number of data sources increases, the time cost of the accurate search increases exponentially, while the time cost of the approximate search is basically not change much because it controls the choice of the data sources.

**Cost.** We compared the cost of the result of the query returned by the accurate search and the approximate search. As the number of available data sources increases, the costs of the results of the two methods are both declining. This is because that the more data sources we can access, the more likely we are tantamount to discover data sources with high-quality and low-cost. Overall, the cost of the accurate search results is substantially lower than that of the approximate search, because the accurate search returns the optimal solution.

**Quality.** The quality of the optimal solution returned by accurate search is usually closer to the quality threshold and therefore lower than the quality of the result of the approximate search. Please note that when the data source is 10, there is no result that satisfies the quality threshold. To comprehensively compare the two methods, when implementing the two methods, we require that both methods return the highest quality result in this case. At this time, the results of the two methods are the same, and the quality score is 0.945.

#### 7.1.2. Varying $|O_{Q}|$

**Figure 6:**
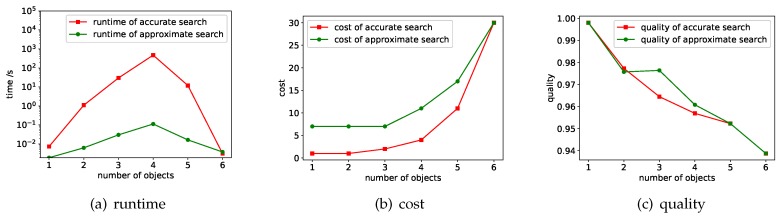
Experimental results when varying $|O_{Q}|$.

When varying $|O_{Q}|$, the relative merits of the two methods in different aspects are the same as the situation of changing |S|, but the trends of runtime, cost and quality are different. We fixed |S| to 56 (i.e., we use all the available data sources) and the quality threshold to 0.95. We change $|O_{Q}|$ from 1 to 6. Figure 6 shows the experimental results.

**Runtime.** Since the runtime of the accurate search varies greatly, the vertical axis of Figure 6a uses a logarithmic scale. As can be seen from the results that the time cost of accurate search is much higher than the approximate search. As $|O_{Q}|$ increases, the runtime increases first and then decreases. This is because when $|O_{Q}|$ is large enough, the result that satisfies the quality threshold is gradually reduced (the quality score is the probability multiplication, thus more observations will make the probability smaller). Based on the pruning strategy of Theorem 1, the search space is also gradually reduced. When $|O_{Q}|=$ 3 and 4, since the combination of sources and values that satisfy the quality threshold will explode, the number of nodes in the search tree generated by accurate search also increases, resulting in a sharp increase in runtime. Meanwhile, since the sources that can be used for the extension of the search tree are pre-constrained in approximate search, the number of search tree nodes increases slower, and the runtime is considerably less than the accurate search.

**Cost.** As $|O_{Q}|$ increases, the cost increases, because in order to find the records of all objects in $O_{Q}$, it is often necessary to access more data sources. Overall, the cost of accurate search is lower than the cost of approximate search, but when $|O_{Q}|=6$, the two are the same, because no result meets the quality threshold at this time, both methods return the result with highest quality score.

**Quallity.** As $|O_{Q}|$ increases, the quality of the results decreases. Accurate search sacrifices quality to reduce costs while ensuring that the quality threshold is satisfied, so sometimes the resulting quality scores are lower than the approximate search. For example, in the case of $|O_{Q}|=3$ or $|O_{Q}|=4$, accurate search can find the solution that with lowest cost, but the quality is also lower than the solution returned by approximate search.

### 7.2. Evaluation of Search Space Size Estimation

**Figure 7:**
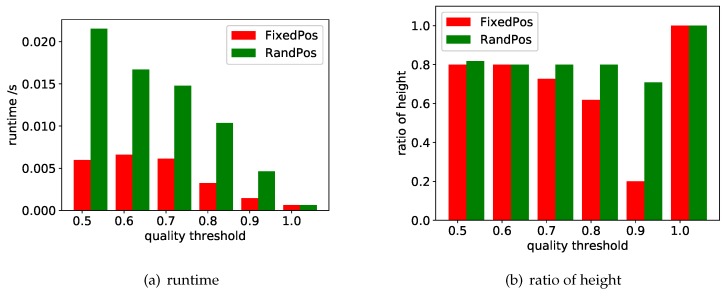
Experimental results of search space size estimation.

The most important step in adjusting the inappropriate quality threshold is to estimate the size of the search space and the height of the search tree. We have experimented with the two estimation strategies (i.e., FixedPos and RandPos) proposed in Section 6.1. The experimental results are shown in Figure 7. In the experiments, |S| and $|O_{Q}|$ are set to be the maximum values (i.e., 56 and 6 respectively), and the quality threshold θ varies from 0.5 to 1.

**Efficiency.** We first compare the runtime of the two strategies. The time cost of RandPos is overall slightly higher than FixedPos. When the threshold changes, the time cost of RandPos and FixedPos decreases(that is, the efficiency rises). This is explained by the fact that the higher the threshold, the earlier the search process stops. At the same time, we found that the time cost of both strategies is much lower than the time cost of accurate search and approximate search. Even if the quality threshold is set very low, the two strategies can complete the entire estimation process within 30 ms. With the same settings, accurate search and approximate search may take tens of minutes to return results. Therefore, the time cost for estimating the size of the search space is negligible compared to the time the query is executed. In other words, if we can perform the estimation before the query starts, we can assume that this will add little extra time cost.

**Effectiveness.** We compare the estimated height and true height of the search tree. The experimental results are shown in Figure 7b, where *ratio of height* is defined as $\hat{h}/h$. Since $\hat{h}\leq h$, the ratio of height is in [0,1]. This value indicates the accuracy of the estimate. It can be observed in the experimental results that the performance of RandPos is relatively stable compared to FixedPos. When the threshold changes, the performance of RandPos is stable (basically kept above 0.8), while FixedPos may fluctuate depending on the fixed position to be replaced. Concretely, some of the branches in the search tree may be pruned very early. For example, some objects may have fewer candidate values, then FixedPos may have no value to replace very early on these objects. Or, some attributes fail to satisfy the quality threshold after a few steps of replacement, so the corresponding branches will be pruned early. When the threshold is set to 0.9, the situation that the replacement of a single location cannot satisfy the quality threshold is very easy to occur, so the estimated accuracy is not stable enough. When the threshold is 1, the search tree height is 1 because there is no query result can meet the threshold, so the height ratio of FixedPos and RandPos is 1, that is, both of the two strategies can accurately estimate the height of the search tree.

In summary, the time overhead of the two strategies is much lower than the execution of the search, and the estimated search tree height is basically close to the real situation. Therefore, if we can perform the estimation operation before the query starts, it can effectively avoid the unreasonable threshold setting while adding little time overhead.

## 8. Conclusions and Future Work

This paper studies the problem of answering MQQ query on multi-sources in sensor-cloud systems. First, a general definition of MQQ answering problem in the multi-source environment is given. After that, the quality measurement is provided based on relative quality constraints, and two methods for solving the MQQ answering problem are proposed. To deal with the case that it is difficult to set the reasonable quality threshold, a method to automatically check the resonablility of the threshold is proposed.

In future work, we will study how to get more types of quality scores of different sources by the methods of data mining or machine learning, and will explore other forms of quality-preserving queries.
